# Development of polymer based cryogel matrix for transportation and storage of mammalian cells

**DOI:** 10.1038/srep41551

**Published:** 2017-01-31

**Authors:** Jyoti Kumari, Ashok Kumar

**Affiliations:** 1 Department of Biological Sciences and Bioengineering, Indian Institute of Technology Kanpur, Kanpur- 208016, UP, India

## Abstract

We studied the potential of polymeric cryogel matrices such as 2-hydroxyethyl methacrylate (HEMA)-agarose (HA) and gelatin matrix as a transporting and storage material for mammalian cells. Both the HA and gelatin matrices were found to possess a homogenous distribution of pores as shown by scanning electron microscopic (SEM) images and flow rate of 8 and 5 mL/min, respectively. In the case of HA cryogel, after 5 days of simulated transportation, C2C12 cells kept in cryogel matrix showed higher percentage viability (89%) as compared to 64.5% viability of cells kept in suspension culture. The cells recovered from the HA cryogel were able to proliferate as revealed by the microscopic analysis. In the case of gelatin cryogel, it was shown that C2C12 cells seeded on the cryogel under simulated transportation condition were found to proliferate over the period of 5 days. It was also observed that the cells after simulation can be cryopreserved and the duration of cryopreservation does not affect their viability. Furthermore, gelatin cryogel was used for cryopreservation of HepG2 and HUVEC cells to extend the system for other cell types. These results show the potential of cryogels as efficient, low-cost transporting matrix at room temperature and in cryo-conditions.

Animal cell culture refers to the culturing of cells derived from higher eukaryote such as mammals, birds and insects. These cells lines are provided by various commercial suppliers and laboratories^1^. One important aspect of animal cell culture technique is the transportation and storage of cells. Cells are often transported from one city to another and also from one country to another^1^. There are many existing methods for cell transportation. The success of these methods is measured through the viability and proliferation capability of the transported and stored cell^2^. The existing methods used for viable cell transportation include using either a T-flask filled with the medium, to avoid damage to a cell^1^, or using a cryovial under cryo-condition using dry ice^1^,^3^. Live cells transportation in T-flask often suffers from cell damage due to shear force, rapid exhaustion of oxygen and change in pH of the medium. Further, it is also associated with limitation in shipping time (usually up to 24 h)^4^. On the other hand, use of methods involving dry ice and liquid nitrogen are costly and require special kind of non-insulated containers to keep cells in frozen condition. Moreover, these conventional methods have several limitations in terms of overall efficiency, immediate processing on arrival and cost^1^,^3^. To overcome these limitations in cell transportation, gel-based transportation systems have been proposed. Different approaches have been explored using these gel based systems. Gels used are prepared using polymers such as agarose and gelatin^1^,^5^. In one of the systems, agarose gel has been developed to transport cells adhered on the culture plate^1^. In another system, for transportation of live cells, encapsulation of cells in gel has been evaluated^5^,^6^. These gels provide a cushion-like support to the cell seeded on a culture plate and thus overcoming some of the above limitations. However, cells transported using these gel based methods need to undergo some processing before they could be used for further cell culture. Therefore, it is highly desirable to look for an alternative method of cell-transportation that could maintain viability of cells during transportation as well as allow their direct use after transportation without further processing^1^,^5^.

Another very important aspect of cell culture technique is cryopreservation of cells. In conventional methods, cryopreservation of cell suspension is done using slow cooling and fast warming rates. However, it does not take into account the difference in response to dehydration, cooling and warming shown by the complex cell system and simple suspension of a cell. Thus, complex cell system shows poor cell recovery after preservation. It has been previously reported that cell in a monolayer is much more prone to cryoinjury compared to cells in suspension^7^,^8^. It is because cell-to-cell and cell-to-matrix interaction make cell much more susceptible to cryoinjury at the time of thawing^9^. To overcome these limitations, gel-based methods are used for cryopreservation through cells encapsulation^8^. This method has an advantage as it protect cells against any kind of mechanical damage and reduce the chance of cell disruption via immobilization of cells within the hydrogel. However, this method cannot be used for every cell-type, because for each cell-type a gel of specific mechanical property is required^10^. Recently, matrices synthesized at subzero temperature using cryogelation technology known as cryogel have been reported for cryopreservation^10^,^11^. These polymeric cryogels possess three-dimensional (3D) structure and have already been used as a scaffold for tissue engineering applications^12^. Cell-scaffold construct for regenerative medicine was cultured *in vitro* which was then transplanted in *in vivo*^11^,^13^,^14^. Pausing or storing of the developed tissue engineered construct is not allowed during this entire process. Therefore, cryopreservation of cell-scaffold construct having viable and functional cells is a preferable approach to meet the demand. Due to their ready-to-use nature, they could be immediately utilized. These cryogel-based methods for cryopreservation have advantages over the gel-based method because in the former cell encapsulation and gel formation do not occur simultaneously^10^.

In this study, we have examined the possibility of using cryogels for cell transportation so that it could be integrated with the methodology of using cryogel for cryopreservation. Our findings suggest that the cells recovered from the cryogel were viable after simulated transportation using 2-hydroxyethyl methacrylate (HEMA)-agarose (HA) cryogel. Further, we have also shown that the cells stored in the gelatin cryogel remain fit for cryopreservation even after the simulated transportation for up to five days. The results show that cryogels not only overcome the above-mentioned problems of transportation of cells but also provide a ready-to-use scaffold for further engineering of adherent cells, including cryopreservation. Using cryogels for transportation and storage one also have an added advantage of cell proliferation during transportation. Cryogels could, therefore, be seen as a better solution for tissue engineering, involving cell-transportation and cell-cryopreservation.

## Results and Discussion

### Synthesis of HA and gelatin cryogels

**Figure 1:**
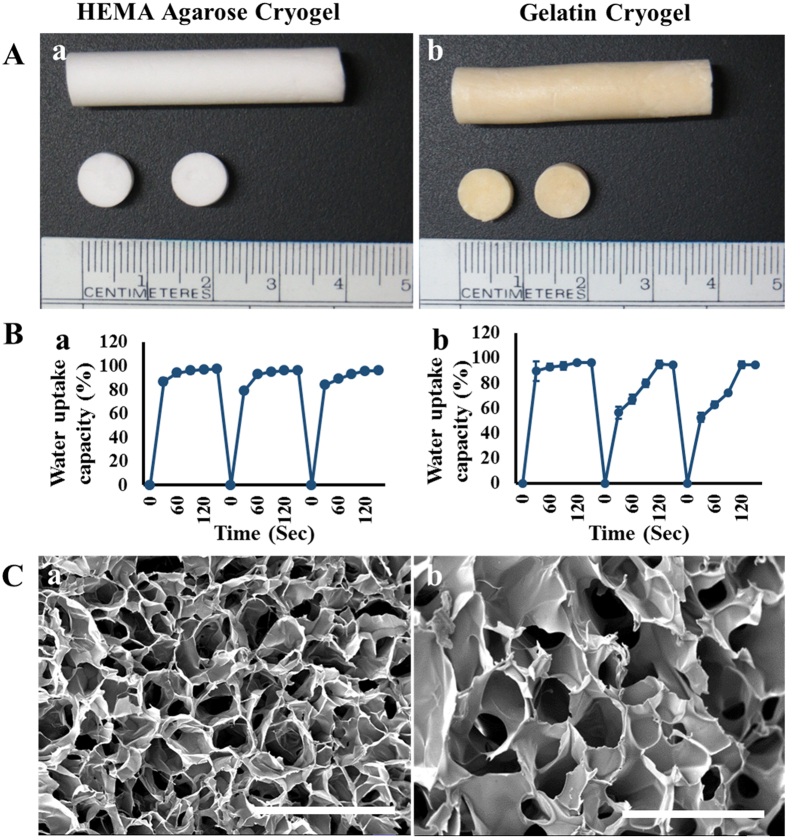
Physical characterization of HA and gelatin cryogels. (**A**) Digital image of (a) HA cryogels and (b) gelatin cryogels. (**B**) Cyclic swelling and de-swelling kinetics of (a) HA cryogels and (b) gelatin cryogels. (**C**) Scanning Electron Microscopy image of (a) HA cryogels and (b) gelatin cryogels. Scale bar (**C**): 200 μm; magnification (**C**): 500×.

Both HA and gelatin cryogels were synthesized via cryogelation process. HA cryogel (Fig. 1Aa) formation involves free radical polymerization of HEMA into polyHEMA^15^ and physical gelation of agarose^16^. Gelatin cryogel (Fig. 1Ab) synthesis involves crosslinking between aldehyde group of glutaraldehyde and the amino group of gelatin molecules^17^. All the three polymers agarose, HEMA and gelatin in different compositions have already been used as cell culture matrix for various tissue engineering purpose such as: chitosan-agarose-gelatin cryogel has been used for cartilage tissue engineering^18^, polyHEMA-gelatin cryogel was used for culturing myoblast cell line (C2C12)^19^ and PEG-alginate-gelatin cryogel has been used for liver tissue formation^20^. In this study, synthesized HA and gelatin cryogels were used for simulated transportation of cells at room temperature. Further, gelatin cryogel was used for cryopreservation of cells for a given time period at −80 °C after simulated transportation.

Rational behind using HEMA and agarose is that since both are hydrophilic polymers^15^,^21^ they do not allow cells to get adhered on their surface, instead they provide support to the cells while transportation. The hydrophilic surface resists protein absorption thus it also resist cell adhesion^21^. Agarose has already been used for encapsulating the cells as a hydrophilic agarose microbeads. Thus in this study, we chose these two given polymers to encapsulate the cells during transportation. Encapsulation provides a 3D environment to the cells and cryogel matrix act as a cushion for cells. The cells recovered from HA cryogel can be further used for various tissue engineering or bioprocess application. Agarose also provided strength to the HA cryogel. On the other hand, gelatin possess amino acid sequence arginine-glycine-aspartic acid (RGD) on its surface and hence provides the adherent surface for cell^19^,^22^. Gelatin had already been used as a matrix for various cells example: neural cells^23^, cartilage^18^ and liver^20^ cells thus gelatin can be used as a common matrix for cryopreservation of various cells. The gelatin used in the study was derived from cold water fish skin. It has been studied that gelatin derived from cold-water fish, has higher amounts of hydrophobic amino acids and low water vapour permeability than warm-water fish gelatin or mammalian gelatin^24^. The hydrophobic surfaces have a strong tendency to absorb protein and thereby bind with cells. Thus, the type of gelatin has additional adherent property to be used as a cell culture matrix.

### Flow rate and swelling kinetics analysis

Flow rate measures the volume of solvent that passes through the cryogel monolith in one min. Flow rate of HA and gelatin cryogels were found to be around 8 mL/min and 5 mL/min, respectively indicating that the pores in the synthesized cryogel were interconnected. In the case of HA cryogel, the high flow rate enable easy recovery of the cells after transportation. On the other hand, in the case of gelatin cryogel good flow rate helps easy passage of nutrient and oxygen during simulated transportation and also makes dimethyl sulfoxide (DMSO) available for all the cells in case of cryopreservation. DMSO is needed for the cells present inside the cryogel as these cells may experience cryoinjury in absence of cryoprotectant.

Swelling kinetics determine the solvent uptake capacity of the cryogels. In the first cycle of swelling-de-swelling, HA cryogels swelled up to 86 ± 2.3% (Fig. 1Ba) while, gelatin cryogel swelled up to 89 ± 8% (Fig. 1Bb) in 30 sec and both the cryogels reached their equilibrium state in 90 sec. During the second cycle, in the case of HA cryogels, there was a slight decrease in initial water uptake capacity rate. However, it reached its equilibrium state in 90 sec. This shows that the behavior of HA cryogels does not change with cyclic swelling and de-swelling process. From the result, we can also infer that the behavior of the HA cryogel in terms of solvent uptake capacity will remain same during cell transportation. The high swelling capacity of the HA cryogel helps in easy recovery of cells without damaging the cryogel matrix. On the other hand, in the case of gelatin cryogels equilibrium was reached in 120 sec in the second cycle. The soft nature of gelatin cryogels might be the reason for this increase in equilibrium time.

### Scanning electron microscope (SEM) analysis

The surface morphological analysis was done using SEM and it showed homogenous pore distribution on both the cryogels. Pore diameter was found in the range of 10–100 μm in the case of HA (Fig. 1Ca) and 20–90 μm in the case of gelatin (Fig. 1Cb). In the case of HA cryogel presence of pores helps in easy recovery of cells. On the other hand, in the case of gelatin cryogel pore wall provides an adherent surface to the cells and helps in cell proliferation.

### Pore interconnectivity and Diffusion analysis

The solute permeability was analyzed by studying the diffusion of BSA through the cryogel. The effective diffusion coefficient (Deff) of BSA in the case of HA and gelatin cryogel were found to be 3.4 × 10^−7^ and 3.8 × 10^−7 ^cm^2^/s, respectively, which are slightly lower than diffusion coefficient in water (Do), i.e. 5.9 × 10^−7 ^cm^2^/s^25^. As the matrices have high porosity and pore-interconnectivity, the diffusion property of cryogel matrices lies in the range of Do in water^14^. This close resemblance of the diffusion coefficient indicates easy passage of BSA through the cryogel matrix. This unhindered movement is advantageous in passing nutrient and oxygen during cell transportation.

### Rheology and compression analysis

Flow and deformation of a materials in response to applied force can be studied by rheology, which includes elasticity, viscosity, and visco-elasticity behavior. Elastic modulus and elastic nature of the material is defined as storage modulus (G′). The dissipation (viscous) of the flow is represented by loss modulus (G″). The visco-elasticity behavior or phase angle is the difference between the storage and loss modulus.

**Figure 2:**
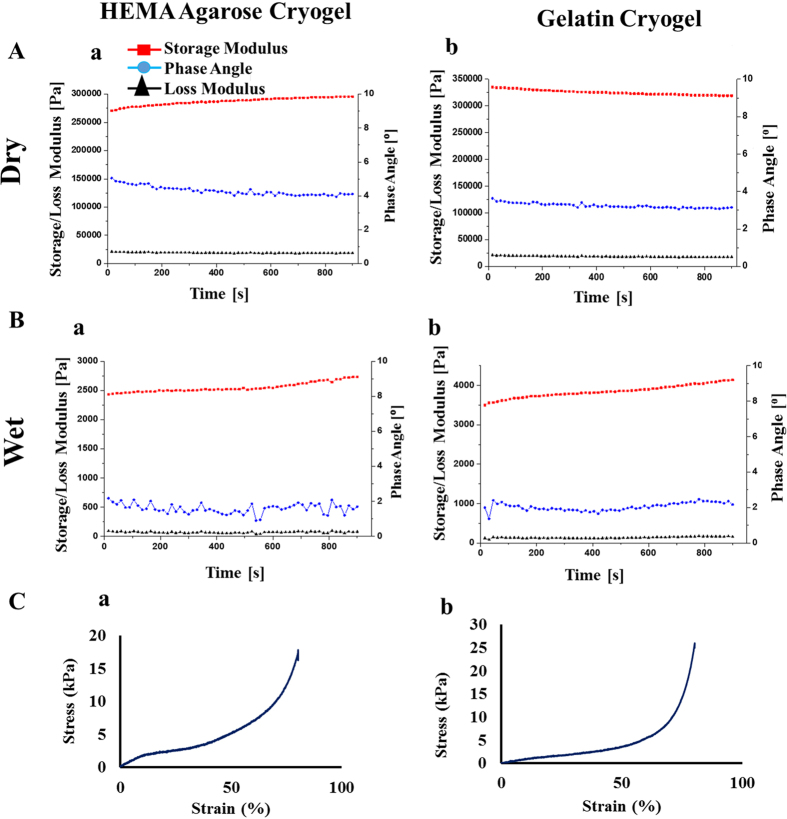
Rheology and compression analysis of HA and gelatin cryogels. (**A**) Rheology analysis of dry (a) HA and (b) gelatin cryogel. (**B**) Rheology analysis of wet (a) HA and (b) gelatin cryogel. (**C**) Stress versus strain curve of (a) HA and (b) gelatin cryogel subjected to compression test.

Rheology analysis of HA cryogel showed that storage modulus of cryogel increased from 2.37 × 10^5^ Pa to 2.59 × 10^5^ Pa in the dry state (Fig. 2Aa) and 2.4 × 10^3^ to 2.7 × 10^3^ Pa, in the wet state (Fig. 2Ba). Similarly, in the case of gelatin cryogel the storage modulus decreased from 3.35 × 10^5^ Pa to 3.19 × 10^5^ Pa in the dry state (Fig. 2Ab) whereas, there were an increase in the storage modulus from 3.5 × 10^3^ to 4.1 × 10^3^ Pa in the wet state (Fig. 2Bb). Increase in storage modulus in the case of wet HA and gelatin cryogel indicate that the elastic property of the cryogel increased with time. The wet state has low storage modulus than the dry state indicating that in the wet form the elasticity of the cryogel decreases, mechanical stability increases and also cryogels respond quickly to the applied stress in wet condition. Reduction in phase angle indicates that the both HA and gelatin cryogel are more elastic and less viscous. The viscoelastic nature of the synthesized cryogel supports its use for *in vitro* cell culture.

Mechanical properties of cryogels were analyzed by its microstructure network. Both HA and gelatin cryogels can bear compression strain of 80% without permanent deformation or mechanical destruction. The mechanical strength of both HA and gelatin cryogels is a very important parameter in cells storage and transportation. Young’s modulus of HA and gelatin cryogels were found to be 6.1 ± 0.46 kPa (Fig. 2Ca) and 4.6 ± 0.44 kPa (Fig. 2Cb). Such low value of Young’s modulus demonstrates the elastic nature of the scaffold and thereby supports the culture of mouse myoblast cell line or hepatic cells proliferation. This result also shows the soft nature of the cryogel. The soft and elastic nature of the synthesized cryogel provides it with appropriate physical property for soft tissue engineering.

### Simulated transportation of cells using HA cryogel

**Figure 3:**
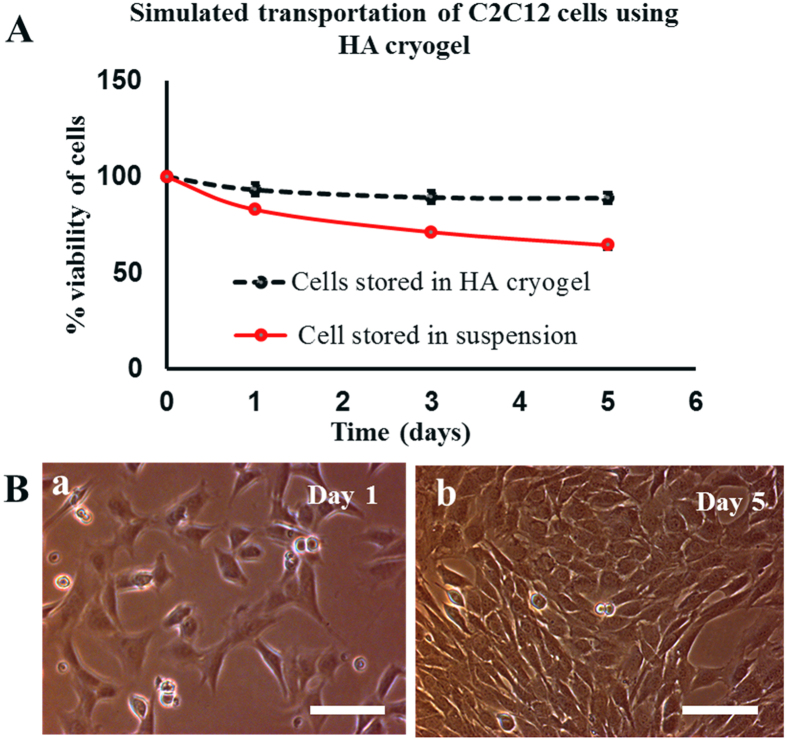
Percentage viability and cell proliferation analysis of C2C12 cells after simulated transportation. (**A**) Percentage viability of recovered C2C12 cells from HA cryogels (test) and cryovial (control) after 1, 3 and 5 days of simulated transportation, (**B**) phase contrast image showing proliferation of recovered C2C12 cells (test) at day 1 (a) and at day 5 (b). Scale bar (**B**): 100 μm; magnification (**B**): 200×.

HA cryogel was used to understand the effect of simulated transportation on the C2C12 cells. For this, cell viability of the cells stored in HA cryogels (test) was compared with the cells viability of cells stored in cryovial as cell suspension (control). Cells were recovered from the HA cryogel matrix using Trypsin EDTA (TE) because the presence of TE helps in disaggregating the cluster of the cells^26^ and also to detach cells from the scaffold surface (if any). It was reported that after five days of simulated transportation, the percentage viability of cells stored in HA cryogels was around 89 ± 2.6% while cells stored in cryovial as suspension showed 64.5 ± 2%. This difference in percentage viability was statistically significant P < 0.05 (Fig. 3A). Cell viability was maintained in case of cells stored in HA cryogels during five days of simulated transportation at room temperature. This enhancement in viability may be due to the reason that the cryogel possibly prevent the shear force and damage produced during simulation and thereby maintained the viability of the cells. Further, recovered C2C12 cells from HA cryogel when cultured on a 24 well plate in a CO_2_ incubator, were growing normally and became confluent in 5 days as revealed by phase contrast microscopy images (Fig. 3B). Therefore it can be concluded that transportation of cells using HA cryogel has an advantage that the cells can be recovered from the cryogel. The recovered cells can be further used for any cell culture application and can be cryopreserved for future use.

In the case of both test and control groups 4-(2-hydroxyethyl)-1-piperazineethanesulfonic acid (HEPES) buffer was added to maintain the change in pH, which may occur due to change in CO_2_ concentration via cellular respiration. HEPES is a better buffering agent than sodium bicarbonate in maintaining pH in cell culture medium. It is known for an effective buffering action even at low temperature^27^. This property of HEPES gives an additional benefit when cells seeded cryogels were stored at room temperature.

### Simulated transportation of cells using gelatin cryogels

**Figure 4:**
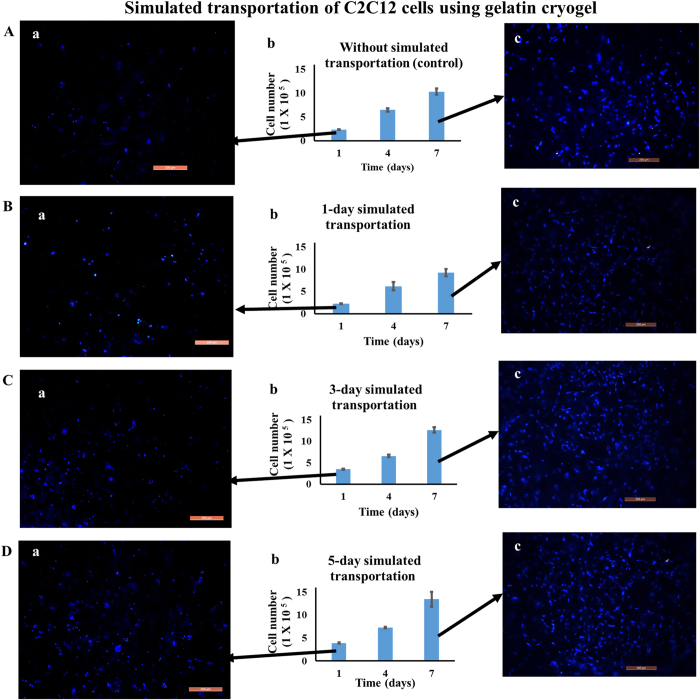
Cell proliferation and fluorescent microscopic analysis of C2C12 cell seeded gelatin cryogel upon simulated transportation. After transportation cells were cultured in a CO_2_ incubator for 7 days (37 °C, 5% CO_2_). Cell number (**A**b) without room temperature storage (control), (**B**b) after 1 day, (**C**b) after 3-day, and (**D**b) after 5-day simulated transportation. Nuclei staining at day 1 incubation (**A**a,**B**a,**C**a,**D**a) and at day 7 incubation (**A**c,**B**c,**C**c,**D**c) in case of control, after 1- day, 3-day, and 5-day simulated transportation, respectively. Nuclei were stained with DAPI (blue). Scale bar: 200 μm; magnification: 100× .

Simulated transportation of C2C12 cells was performed using gelatin cryogel in order to develop a ready-to-use scaffold. Effects of simulated transportation on cell viability and proliferation of cells were studied by storing cell-laden cryogels at room temperature for one, three and five days. Gelatin cryogel seeded with 1 × 10^5^ cells after one day simulated transportation showed the presence of 2.2 ± 0.07 × 10^5^ viable cells. Comparing this with the positive control, cell seeded gelatin scaffold incubated directly in CO_2_ incubator, show no significant difference in number of cells (Fig. 4Ab and Bb). However, by the 3^rd^ and 5^th^ day of room temperature incubation, the number of cells on the scaffold was found to be around 3.5 ± 0.117 × 10^5^ and 3.9 ± 0.136 × 10^5^, respectively (Fig. 4Cb and Db). This indicates that cell number increased over five days and thereby, showing cells proliferation during simulated transportation.

Further, in order to check the proliferation capability of the cells-laden cryogel after simulated transportation, they were cultured in a CO_2_ incubator for 7 days. As HEPES lowered the pH of the medium in a CO_2_ incubator, scaffolds were washed to remove HEPES buffer before transferring it into a CO_2_ incubator^27^. After seven days of incubation, there was a 4-fold increase in cell number for the positive control (without simulation) and for one day simulated transportation experiment (Fig. 4Ab and Bb). However, in the case of three and five days of simulated transportation experiment, cell number increased by 3.5-fold and 3.4-fold after 7 days of incubation, respectively (Fig. 4Cb and Db). This difference may be due to the stress condition experienced by the cells during simulated transportation. Fluorescent image of day 1 showed the presence of cells after simulated transportation (Fig. 4Aa,Ba,Ca and Da). Increase in cell number from day 1 to day 7 showed that the cells were proliferating. (Fig. 4Ac,Bc,Cc and Dc). The microscopy results further confirmed that simulated transportation cells were viable and able to proliferate when cultured in a CO_2_ incubator.

### Integrated system of cell transportation and cryopreservation

**Figure 5:**
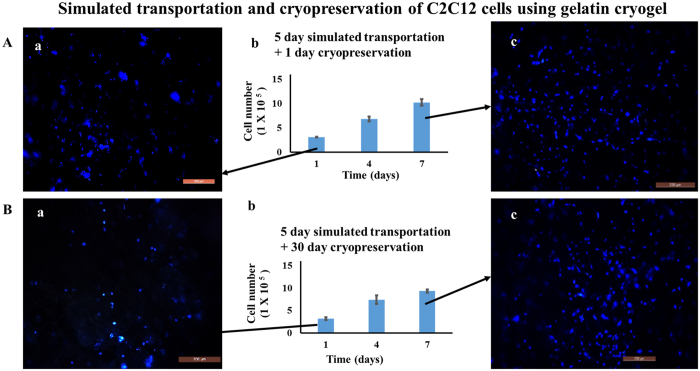
Cell proliferation and fluorescent microscopic images of C2C12 cells seeded on the gelatin cryogels kept at room temperature over the time period of 5 days followed by cryopreservation. Post thawing cells were cultured in a CO_2_ incubator for 7 days (37 °C, 5% CO_2_). Cell number (**A** and **B**) after 5 day room temperature storage followed by (**A**b) 1 day and (**B**b) 1 month of cryopreservation. Nuclei staining at day 1 (**A**a,**B**a) and at day 7 (**A**c,**B**c) after 1- day, and 1-month of cryopreservation, respectively. Nuclei were stained with DAPI (blue). Scale bar: 200 μm; magnification 100×.

From the above experiments we found that after five days of simulated transportation using HA and gelatin cryogel, cells retain their viability. Further, we also tried to understand whether the viable cells recovered after simulated transportation can be cryopreserved or not. For this, after five days of simulated transportation of cell-laden gelatin cryogels, was cryopreserved for 1 day and 30 days. The viable cells recovered after cryopreservation were compared with the initial number of cells prior to five days of simulated transportation. It was found that after five days of simulated transportation followed by 1 day and 30 days of cryopreservation 78 ± 1.7% and 81 ± 7.4% of viable cells were recovered, respectively (Fig. 5Ab and Bb). Cell viability was maintained prior to cryopreservation, as the 3D porous structure of cryogel helps in inhibiting the stress developed at the time of freezing and thawing. This also shows that duration of cryopreservation does not have any significant effect on the viability of post-thawed recovered cells.

Further, we have also checked the proliferation behavior of the viable cells recovered after five days of simulated transportation followed by cryopreservation for 1 day and 30 days. For this, after thawing cells seeded cryogels were kept in a CO_2_ incubator for a period of 7 days. After seven days of incubation, there was 2.9- and 3.3-fold increase in cell number, in the case of 1 day and 30 day cryopreservation, respectively (Fig. 5Ab and Bb). However, in the case of control (5 days simulated transportation and without cryopreservation), there was a 3.4-fold increase in cell number (Fig. 4Db). Increase in value of absorbance in a CO_2_ incubator from day one to day seven shows that cells seeded on the cryogel were functional and proliferating well. This was further substantiated by fluorescent imaging using a nuclear stain, DAPI (Fig. 5Aa,Ac,Ba and Bc). This shows that cryopreservation does not affect morphology and proliferation behavior of cells seeded gelatin cryogel.

Here in our system, cells were adhered on the surface of cryogel matrix while simulated transportation and cryopreservation. As the porous structure of cryogel lessen the stress produced while freezing and thawing, the viability of cells was maintained. Transportation and cryopreservation of cells using cryogel matrix have an added advantage, as in this case detachment of the cells from the matrix is not needed. Therefore one can directly use the cells seeded scaffold after 5 days of transportation and can also freeze the scaffold for later use.

### Effect of cryopreservation on HepG2 and HUVECs cells

**Figure 6:**
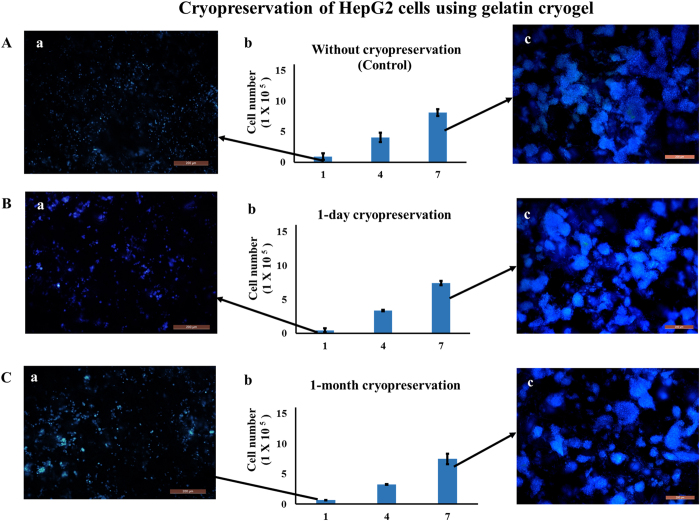
Cell proliferation assay and fluorescent microscopic images of HepG2 cells seeded on gelatin cryogel. Post thawing cells were cultured in a CO_2_ incubator for 7 days (37 °C, 5% CO_2_). Cell proliferation assay of cells seeded gelatin cryogel (**A**b) without cryopreservation (control), (**B**b) 1 day and (**C**b) 1 month cryopreservation. Nuclei staining at day 1 (**A**a,**B**a,**C**a) and at day 7 (**A**c,**B**c,**C**c) in case of control, after 1-day, and 1-month cryopreservation, respectively. Nuclei were stained with DAPI (blue). Scale bar: 200 μm; magnification: 100×.

In order to understand how the developed system behave with other cell types, we have checked the effect of cryopreservation on the viability of HepG2 and HUVECs cells using gelatin cryogel. Proliferation behavior of HepG2 cells seeded on gelatin cryogel was monitored for 1 day and 30 days duration of cryopreservation. There was no significant difference observed in viability and proliferation behavior of cells stored at cryo condition for 1 day and 30 days (P > 0.05) (Fig. 6Ab,Bb and Cb). Similarly, fluorescent image result showed a similar type of proliferation profile in case of 1 day (Fig. 6Aa,Ba and Ca) and 30 days cryopreservation (Fig. 6Ac,Bc and Cc). Cryopreservation for a longer duration, that is 30 days, does not have any adverse effect on cells proliferation. This denotes that these cryogels can be used as an alternate method of cell cryopreservation, having the advantage that the cell can be used as *in vitro* and *in vivo* 3D cell culture applications even after 30 days of cryopreservation.

**Figure 7:**
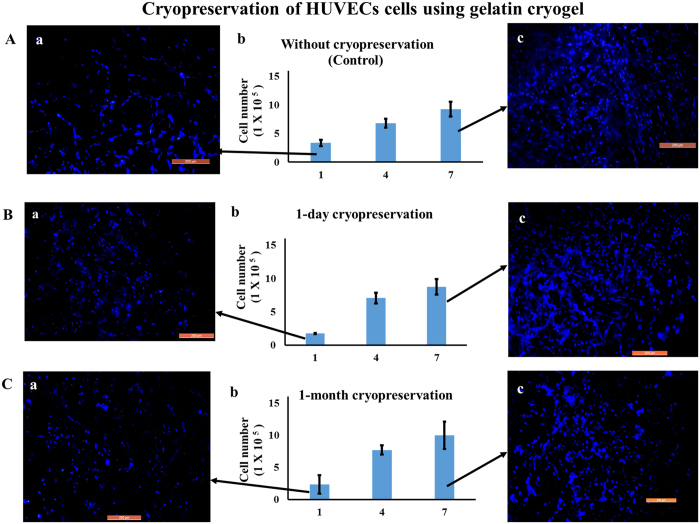
Cell proliferation and fluorescent microscopic images of HUVECs cells seeded on gelatin cryogel. Post thawing cells were cultured in a CO_2_ incubator for 7 days (37 °C, 5% CO_2_). Cell proliferation assay of cells seeded gelatin cryogel (**A**b) without cryopreservation (control), (**B**b) 1 day and (**C**b) 1 month cryopreservation. Nuclei staining at day 1 (**A**a,**B**a,**C**a) and at day 7 (**A**c,**B**c,**C**c) in case of control, after 1-day, and 1-month cryopreservation, respectively. Nuclei were stained with DAPI (blue). Scale bar: 200 μm; magnification: 100×.

In the case of HUVECs-seeded gelatin cryogel, after 1-day cryopreservation followed by 1-day incubation in a CO_2_ incubator, the absorbance value showed a significant difference as compared to positive control (without cryopreservation) (P < 0.05) (Fig. 7Aa and Ab). However, upon 4 and 7 days CO_2_ incubation there was no significant difference as compared to positive control (without cryopreservation) (P > 0.05) (Fig. 7Aa and Ab). On the other hand, in case of 30 day cryopreservation, after 1, 4 and 7 day CO_2_ incubation there was no significant difference found as compared to positive control and 1 day cryopreservation. Fluorescent microscopic results showed an increase in cell number from day 1 to day 7 CO_2_ incubation. This further substantiate that proliferation behavior of post-thawed recovered cells did not alter with the duration of cryopreservation and was able to grow and proliferate.

### Cell viability and proliferation assay

Cell viability was measured using two different approach based on surface property of the cryogel matrices. In case of HA cryogel, cells can be eluted after transportation thus, cell viability of eluted cells were measured using trypan blue. However, in case of gelatin cryogel it is difficult to isolate the cells as cells were adhered on the polymer surface thus we have used MTT assay to measure viable cells. Here we are not comparing the % viability results among the two cryogel systems as both the cryogel were meant for different application. In both the cryogel we observed cells were viable and able to proliferate after simulated transportation. Further in case of gelatin cryogel we have also observed that cells were able to proliferate after cryopreservation.

In summary, we have explored the potential of HA and gelatin cryogel to transport and store adherent-cells. As both the cryogel have different surface properties, they can be used for various application. We have synthesized HA cryogel for cell’s transportation in which cells can be eluted after transportation. On the other hand, gelatin cryogel can be used for both cell transportation as well as for cryopreservation. In both the cryogels we observed cells were viable and able to proliferate after simulated transportation. Using HA cryogels and C2C12 cells, we have demonstrated that the adherent-cells kept in cryogels remain up to 25% more viable as compared to the adherent-cells kept in cryovial as suspension culture under the same simulation conditions for transportation. We have also shown that by using gelatin cryogels and C2C12 cells, the adherent-cells can be stored in cryogels for up to five days at room temperature while retaining up to 80% viability. It has further shown that these adherent-cells indeed remain fit for cryopreservation even after the simulated transportation in cryogels for up to five days. This suggests that cryogels can minimize the effect of shear force, which is the main cause for the reduction of viability in transporting cells in suspension. Above results clearly show the potential of cryogels as an efficient, low-cost storing and transporting matrix at room temperature and at cryo-conditions, and therefore may have important implications for ready-to-use transplantation, *in vitro* drug testing, and regenerative medicines. These results thus pave the way for establishing cryogels as a better solution for tissue engineering, involving cell-storage, cell-transportation, and cell-cryopreservation.

## Material and Methods

### Materials

Gelatin (from cold water fish skin), HEMA, 97%, poly(ethylene glycol) diacrylate (PEGDA), Dulbecco’s modified Eagle’s medium (DMEM), 3-(4,5-dimethylthiazol-2-yl)-2,5-diphenyl tetrazolium bromide (MTT, 98%), TE, 4′-6-diamidino-2-phenylindole (*DAPI*), and β-endothelial cell growth factor (human) were purchased from Sigma Chemical Co. (St. Louis, MO, USA). Agarose (low EEO) and HEPES were obtained from Qualigens Fine Chemicals (Mumbai, India). Glutaraldehyde (25% in aqueous solution) and DMSO were purchased from Loba Chemie (Mumbai, India). Fetal bovine serum (FBS) and streptomycin-penicillin antibiotic solutions were bought from HyClone (Utah, USA). N,N,N′,N′-tetramethyleneethylenediamine (TEMED) was obtained from Bio-Rad Labs (Hercules, CA, United States) and a*mmonium persulfate* (APS) was from Merck Limited (Mumbai, India). All other chemicals used were of analytical grade.

### Synthesis of HA and gelatin cryogel

HA and gelatin cryogel were synthesized using cryogelation technology. In the case of HA cryogel synthesis, agarose solution (1% w/v) was prepared by dissolving 100 mg agarose in 9.8 mL of degassed water. To this solution, HEMA (100 μL) with the final concentration of 1% (v/v) was added followed by the addition of polyethylene glycol-diacrylate PEG-DA (ratio 1:2, PEG-DA: pHEMA), APS (0.1 mL of 10% w/v) and TEMED (10 μL). For gelatin cryogel, gelatin (5% w/v) was dissolved in 9.9 mL of deionized water and pre-cooled at 4 °C for 15 min. Crosslinking agent glutaraldehyde [1% (v/v)] was added to solution. Both the solutions were then poured into a syringe and were placed in a cryostat (Julabo, Seelbach, Germany) at −12 °C for incubation of 16 h. After incubation, polymerized matrices were thawed using de-ioinized water at room temperature and then vacuum-dried for storage.

### Flow rate analysis

A cryogel monolith of 12 mm height and 8 mm diameter was used to determine the flow rate using a peristaltic pump. Deionized water was allowed to pass through cryogel monolith at varying flow rate ranging from 1 to 8 mL/min until there was no back-pressure generated in the cryogels. In a separate experiment as control, the flow rate was determined without connecting a cryogel in between the flow path^28^.

### Cyclic swelling kinetics and morphological analysis

Swelling kinetics was determined using a conventional gravimetric procedure, as described earlier^29^,^30^. Three cryogel discs of equal size (2 mm thickness and 8 mm diameter) and weight were dipped in 0.1 M PBS and weighed at regular time interval. Swelled cryogels were then dried for 24 h and de-swelling were done up to three consecutive days with the same cryogel sample. The percentage of water uptake capacity (W_u,_%) was calculated using the formula


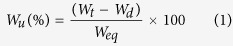


where, W_t_, W_d_ and W_eq_ is the weight of disc at regular time interval, dry disc and swollen disc at swelling equilibrium respectively.

The morphology and pore size of the synthesized cryogel was determined using scanning electron microscope (SEM) (FEI Quanta 200). For this wet cryogel samples were dehydrated using gradient ethanol (20–100%) and then dried under vacuum. Samples were then coated with gold using sputter gold coater machine (Vacuum Tech, Bangalore, India) and observed under SEM.

### Pore interconnectivity and diffusion analysis

Diffusion of bovine serum albumin (BSA, Mw: 67,000 kDa) was used to analyze the pore interconnectivity of HA and gelatin cryogel by comparing the D_eff_ of BSA in the scaffolds to their diffusion coefficient in free water (D_0_). The cryogel disc was cut into 5 mm height (8 mm diameter) and dipped in 5 mg/mL BSA solution prepared in 10 mM PBS (pH 7.2, 0.02% sodium azide). The scaffolds were incubated at 37 °C for 96 h and were allowed protein solution to equilibrate throughout the scaffolds. The externally adsorbed proteins were washed away with PBS and the scaffolds were then immersed in 10 ml of PBS at 37 °C. Periodically, aliquots of 1 ml were collected and replaced with fresh PBS^31^. The protein concentration of the aliquots was measured using a Pierce^®^ BCA protein assay kit according to the manufacturer’s instructions. Based on free volume theory, Deff was estimated using the following formula valid for monolithic devices^32^.


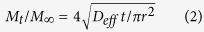


Where, Mt is the cumulative amount of protein released at time t, M∞ is the amount of protein originally present in the scaffold and r is the radius of the scaffold.

### Rheology and compression analysis

The rheological analysis of both the cryogel samples was done using Anton-Paar MCR 102 rheometer. Cryogel samples were cut into the size of 2 mm thickness (8 mm diameter) and placed under load cell of rheometer set at 37 °C. Analysis of samples was carried out at a load of 1 N and frequency of 1 Hz in both dry and wet state. The readings were recorded at an interval of 15 s for a total of 15 min.

The compression analysis of swollen HA and gelatin cryogels were performed using uniaxial compression test by the mechanical tester (NI DAQ card USB 6009 with labview software and load cell from Eltek). The analysis was done using scaffolds with dimension 1 cm height and 8 mm diameter by placing the scaffolds in between the two arms of load frame. The scaffolds were then compressed up to 80% of the total length with a speed of 1 mm/min. The applied force and change in column length due to compression was measured. The Young’s modulus was calculated using the equation


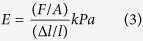


where E is the Young’s modulus, F is the applied force, A is the cross-sectional area, l is the initial length of the test sample and Δl is the change in length under the compressive force.

### Cell culture and cell seeding

Mouse myoblast cell line, C2C12, and human hepatocellular liver carcinoma cell line, HepG2 cells were obtained from National Centre for Cell Sciences (NCCS), (Pune, India). Whereas, human umbilical vein endothelial cells (HUVECs), were a kind gift by Dr. Harishkumar Madhyastha from University of Miyazaki, Japan. C2C12 and HepG2, cells were cultured in DMEM, 10% FBS and 1% w/v penicillin/streptomycin. On the other hand, HUVECs cells were cultured in M199 medium in presence of 20% FBS, and 0.3 ng/mL of a human recombinant β-endothelial cell growth factor. At the time of seeding, cells growing in a T-Flask were trypsinized and the viability of cells was measured using trypan blue.

Prior to cell seeding, monoliths of cryogels were cut into discs of size 2 mm in thickness and 8 mm in diameter. They were then sterilized by gradient ethanol (20%, 40%, 60%, 80%, 100%) followed by three times phosphate buffered saline (PBS) wash of 15 min each. After PBS wash, cryogels discs were incubated in complete medium overnight at 37 °C in a CO_2_ incubator.

### HA cryogel: a non adherent matrix for transportation of cells

Simulated transportation of C2C12 cells were performed using HA cryogel in a cryovial. Cells needed for simulated transportation were suspended in complete DMEM containing 10 mM HEPES buffer. Cell suspension (100 μL) with a total of 1 × 10^5^ viable cells were seeded on the HA cryogel matrix kept in a cryovial (test) and were suspended directly in a cryovial (control). These cryovials were locked using parafilm, wrapped in an aluminum foil, sealed in a zip-lock bag and then transferred into a styrofoam box to prevent any kind of contamination. In order to simulate transportation condition, cryovials were placed on a seesaw rocker for five days at room temperature. Cryovials from both the test and control group were taken out after first, second and fifth day of simulation. The cells from the cryogel were recovered, and the percentage cell-viability of both the groups were compared.

For recovery, in the case of test group cell-laden cryogels from a cryovial were transferred into a syringe and were washed with PBS. TE was then added to the cryogels. After 2 min incubation with TE, complete DMEM was passed through the cryogel and flow through was collected. The recovered cells were then centrifuged and the pellet was dissolved in the medium. In the case of control group, cells in suspension were resuspended in the medium after centrifugation. In both the groups, the percentage viability of the cells was then determined directly by counting live and dead cells using trypan blue.

### Gelatin cryogel: an adherent matrix for cell transportation and cryopreservation

Potential of gelatin cryogel scaffold for cell storage and transportation was evaluated under different conditions. In the first condition, each gelatin scaffold kept in a multiwell plate was seeded with 1 × 10^5^ C2C12 cells and cultured in an incubator for 4 h. After incubation, complete DMEM (200 μL) containing 10 mM HEPES buffer was added on to it. The plates were locked and simulated transportation condition was maintained for one, three and five days, as mentioned above in the case of cryovial. In the case of first condition, at each day point, a plate was taken out and kept in a CO_2_ incubator after washing with PBS. As a positive control, a plate containing cell-seeded cryogels without room temperature incubation was also kept in a CO_2_ incubator. Cell viability and proliferation analysis were done up-to seven days prior to simulated transportation.

In the second condition, after five days of room temperature storage, the cells seeded scaffolds were cryopreserved for 1 and 30 days, after washing with PBS. For cryopreservation, freezing medium (ratio 7:2:1, DMEM:FBS:DMSO) was added on to the scaffolds and plates were sealed using parafilm. After sealing, plates were kept at 4 °C for 10 min and then stored in a styrofoam box at −80 °C. Plates were removed from the styrofoam box and thawed at 37 °C in an incubator. Cryogel scaffolds were washed with PBS twice to remove DMSO followed by the addition of complete medium. For both the conditions cell viability, proliferation and fluorescent image analysis were done up-to seven days.

### Cryopreservation of HepG2 and HUVEC cells on gelatin cryogel

In this experiment gelatin cryogels were seeded with HepG2 cells, kept in a multiwell plate. Cell seeded cryogels were cryopreserved for 1 day and 30 days. The procedure of cryopreservation of cell seeded cryogels was same as mentioned above. After cryopreservation, the proliferation analysis and the imaging were done. A similar set of experiments was also conducted using HUVEC cells.

### Cell viability, proliferation and microscopic assay

Two different test methods were used for analysing the % viability in case of HA and gelatin cryogel based on different application. In the case of HA cryogel, cells were recovered from the cryogel and percentage viability was calculated using trypan blue, a vital stain. The recovered cells were mixed with trypan blue in (1:1) ratio and were counted using a hemocytometer. Percent viability was evaluated using the following formula


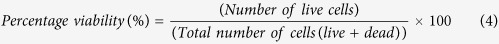


On the other hand, in the case of gelatin cryogel, number of viable cells was determined using MTT assay. MTT assay was performed as reported earlier^33^. Briefly, the medium was aspirated from the well plate containing scaffolds and was washed with PBS. MTT (0.5 mg/mL dissolved in DMEM) solution of 500 μL was added to the scaffolds in the well plate. The plate was then incubated for 4 h at 37 °C. After incubation, MTT was removed and formed formazan crystal was dissolved in 1.5 mL of DMSO. The plate was again incubated for 20 min and sample was collected, and centrifuged at 370 rcf for 2 min. Spectrophotometer (Thermo scientific-Helios Alpha) was used to take a reading of the sample at two wavelengths 570 nm and 630 nm. As the MTT absorbance value directly indicates the cell number, a standard plot was plotted using a known cell number corresponding to the MTT absorbance value. Then using this standard graph MTT absorbance value was converted into cell number^20^.

Cell distribution on the cryogel was analyzed using fluorescent imaging. For staining, cells seeded cryogel were washed with PBS and then fixed with 4% paraformaldehyde for 30 min followed by permeabilization using Triton-X. Sections were then stained using a nuclear stain DAPI and analyzed under a fluorescent microscope.

### Statistical analysis

Data were expressed as mean value ± standard deviation (S.D). P value ≤ 0.05 was considered to be significant. The mean values of the two groups were compared using unpaired t-test.

## Additional Information

**How to cite this article:** Kumari, J. and Kumar, A. Development of polymer based cryogel matrix for transportation and storage of mammalian cells. *Sci. Rep.* **7**, 41551; doi: 10.1038/srep41551 (2017).

**Publisher's note:** Springer Nature remains neutral with regard to jurisdictional claims in published maps and institutional affiliations.

## References

[b1] Yang L., Li C., Chen L. & Li Z. An agarose-gel based method for transporting cell lines. Curr Chem Genomics 3, 50–53 (2009). 20161836 10.2174/1875397300903010050 PMC2802760

[b2] Ebertz S. L. & McGann L. E. Cryoinjury in endothelial cell monolayers. Cryobiology 49, 37–44 (2004). 15265715 10.1016/j.cryobiol.2004.04.003

[b3] Rainov N. G., Trümpler C., Quinones A., Spear M. A. & Kramm C. M. Improved method for transport of living cell cultures. Biotechnology letters 22, 383–385 (2000).

[b4] Betensky R. A. . Shipment impairs lymphocyte proliferative responses to microbial antigens. Clinical and diagnostic laboratory immunology 7, 759–763 (2000). 10973450 10.1128/cdli.7.5.759-763.2000 PMC95951

[b5] Ohyabu Y., Hatayama H. & Yunoki S. Evaluation of gelatin hydrogel as a potential carrier for cell transportation. Journal of bioscience and bioengineering 118, 112–115 (2014). 24457148 10.1016/j.jbiosc.2013.12.005

[b6] Konno T. & Ishihara K. Temporal and spatially controllable cell encapsulation using a water-soluble phospholipid polymer with phenylboronic acid moiety. Biomaterials 28, 1770–1777 (2007). 17215037 10.1016/j.biomaterials.2006.12.017

[b7] Baust J. M. Properties of cells and tissues influencing preservation outcome: Molecular basis of preservation-induced cell death. (Boca Raton: CRC Press, 2007).

[b8] Malpique R., Ehrhart F., Katsen-Globa A., Zimmermann H. & Alves P. M. Cryopreservation of adherent cells: strategies to improve cell viability and function after thawing. Tissue Engineering Part C: Methods 15, 373–386 (2009). 19196129 10.1089/ten.tec.2008.0410

[b9] Acker J., Larese A., Yang H., Petrenko A. & McGann L. Intracellular ice formation is affected by cell interactions. Cryobiology 38, 363–371 (1999). 10413578 10.1006/cryo.1999.2179

[b10] Vrana N. E. . Cell encapsulation and cryostorage in PVA–gelatin cryogels: incorporation of carboxylated ε‐poly‐L‐lysine as cryoprotectant. Journal of tissue engineering and regenerative medicine 6, 280–290 (2012). 21706775 10.1002/term.431

[b11] Katsen-Globa A. . Towards ready-to-use 3-D scaffolds for regenerative medicine: adhesion-based cryopreservation of human mesenchymal stem cells attached and spread within alginate–gelatin cryogel scaffolds. Journal of Materials Science: Materials in Medicine 25, 857–871 (2014). 24297514 10.1007/s10856-013-5108-x PMC3942626

[b12] Mishra R. . Study of *in Vitro* and *in Vivo* Bone Formation in Composite Cryogels and the Influence of Electrical Stimulation. International journal of biological sciences 11, 1325–1336 (2015). 26535027 10.7150/ijbs.13139 PMC4624309

[b13] Umemura E. . Viable cryopreserving tissue-engineered cell-biomaterial for cell banking therapy in an effective cryoprotectant. Tissue Engineering Part C: Methods 17, 799–807 (2011). 21517691 10.1089/ten.tec.2011.0003

[b14] Sarkar J. & Kumar A. Thermo-responsive polymer aided spheroid culture in cryogel based platform for high throughput drug screening. Analyst 141, 2553–2567 (2016). 27027476 10.1039/c6an00356g

[b15] Han Y. A., Lee E. M. & Ji B. C. The physical properties of poly (2-hydroxyethyl methacrylate) copolymer hydrogels used as intravaginal rings. Chinese Journal of Polymer Science 27, 359–366 (2009).

[b16] Tako M. & Nakamura S. Gelation mechanism of agarose. Carbohydrate research 180, 277–284 (1988).

[b17] Farris S., Song J. & Huang Q. Alternative reaction mechanism for the cross-linking of gelatin with glutaraldehyde. Journal of agricultural and food chemistry 58, 998–1003 (2009). 10.1021/jf9031603 20043635

[b18] Gupta A. . Evaluation of three-dimensional chitosan-agarose-gelatin cryogel scaffold for the repair of subchondral cartilage defects: an *in vivo* study in a rabbit model. Tissue Engineering Part A 20, 3101–3111 (2014). 24846199 10.1089/ten.tea.2013.0702 PMC4259173

[b19] Singh D., Nayak V. & Kumar A. Proliferation of myoblast skeletal cells on three-dimensional supermacroporous cryogels. Int J Biol Sci 6, 371–381 (2010). 20617130 10.7150/ijbs.6.371 PMC2899455

[b20] Kumari J., Karande A. A. & Kumar A. Combined Effect of Cryogel Matrix and Temperature-Reversible Soluble–Insoluble Polymer for the Development of *in Vitro* Human Liver Tissue. ACS applied materials & interfaces 8, 264–277 (2016). 26654271 10.1021/acsami.5b08607

[b21] Smith B. H. . Hydrophilic agarose macrobead cultures select for outgrowth of carcinoma cell populations that can restrict tumor growth. Cancer research 71, 725–735 (2011). 21266362 10.1158/0008-5472.CAN-10-2258

[b22] Kozlov P. & Burdygina G. The structure and properties of solid gelatin and the principles of their modification. Polymer 24, 651–666 (1983).

[b23] Vishnoi T. & Kumar A. Conducting cryogel scaffold as a potential biomaterial for cell stimulation and proliferation. Journal of Materials Science: Materials in Medicine 24, 447–459 (2013). 23124526 10.1007/s10856-012-4795-z

[b24] Gómez-Guillén M., Giménez B., López-Caballero M. a. & Montero M. Functional and bioactive properties of collagen and gelatin from alternative sources: A review. Food Hydrocolloids 25, 1813–1827 (2011).

[b25] Burczak K., Fujisato T., Hatada M. & Ikada Y. Protein permeation through poly (vinyl alcohol) hydrogel membranes. Biomaterials 15, 231–238 (1994). 8199296 10.1016/0142-9612(94)90072-8

[b26] Edwards J. & CAMPBELL J. A. The Aggregartion of Trypsinized Bhk21 Cells. Journal of cell science 8, 53–71 (1971). 5102668 10.1242/jcs.8.1.53

[b27] Baicu S. C. & Taylor M. J. Acid–base buffering in organ preservation solutions as a function of temperature: new parameters for comparing buffer capacity and efficiency. Cryobiology 45, 33–48 (2002). 12445548 10.1016/s0011-2240(02)00104-9

[b28] Kathuria N., Tripathi A., Kar K. K. & Kumar A. Synthesis and characterization of elastic and macroporous chitosan–gelatin cryogels for tissue engineering. Acta Biomaterialia 5, 406–418 (2009). 18701361 10.1016/j.actbio.2008.07.009

[b29] Xue W., Champ S., Huglin M. B. & Jones T. G. Rapid swelling and deswelling in cryogels of crosslinked poly (N-isopropylacrylamide-co-acrylic acid). European polymer journal 40, 467–476 (2004).

[b30] Srivastava A., Jain E. & Kumar A. The physical characterization of supermacroporous poly (N-isopropylacrylamide) cryogel: mechanical strength and swelling/de-swelling kinetics. Materials Science and Engineering: A 464, 93–100 (2007).

[b31] Lévesque S. G., Lim R. M. & Shoichet M. S. Macroporous interconnected dextran scaffolds of controlled porosity for tissue-engineering applications. Biomaterials 26, 7436–7446 (2005). 16023718 10.1016/j.biomaterials.2005.05.054

[b32] Hennink W., Franssen O., van Dijk-Wolthuis W. & Talsma H. Dextran hydrogels for the controlled release of proteins. Journal of controlled release 48, 107–114 (1997).

[b33] Mosmann T. Rapid colorimetric assay for cellular growth and survival: application to proliferation and cytotoxicity assays. Journal of immunological methods 65, 55–63 (1983). 6606682 10.1016/0022-1759(83)90303-4

